# New Mesoporous Silica Materials Loaded with Polyphenols: Caffeic Acid, Ferulic Acid and p-Coumaric Acid as Dietary Supplements for Oral Administration

**DOI:** 10.3390/ma15227982

**Published:** 2022-11-11

**Authors:** Gabriela Petrisor, Ludmila Motelica, Denisa Ficai, Roxana Doina Trusca, Vasile-Adrian Surdu, Georgeta Voicu, Ovidiu Cristian Oprea, Anton Ficai, Ecaterina Andronescu

**Affiliations:** 1 Science and Engineering of Oxide Materials and Nanomaterials, Faculty of Chemical Engineering and Biotechnologies, University Politehnica of Bucharest, Gh. Polizu 1-7, 011061 Bucharest, Romania; 2 National Research Center for Food Safety, University Politehnica of Bucharest, Splaiul Independentei 313, 060042 Bucharest, Romania; 3 National Center for Micro and Nanomaterials, University Politehnica of Bucharest, Splaiul Independentei 313, 060042 Bucharest, Romania; 4 Department of Inorganic Chemistry, Physical Chemistry and Electrochemistry, Faculty of Chemical Engineering and Biotechnologies, University Politehnica of Bucharest, Gh. Polizu 1-7, 011061 Bucharest, Romania; 5 Academy of Romanian Scientists, Ilfov Street 3, 050044 Bucharest, Romania

**Keywords:** mesoporous silica, drug delivery, caffeic acid, trans-ferulic acid, p-coumaric acid

## Abstract

In this study, two types of mesoporous silica with different pore structures and volumes were synthesized by the soft-templating method. The two types of mesoporous silica, type MCM-41 and MCM-48, were loaded with three polyphenols—caffeic acid, p-coumaric acid and trans-ferulic acid—in the same ratio of mesoporous silica:polyphenol (1:0.4 *w*/*w*). The materials obtained were characterized from a morphological and structural point of view through different analysis techniques. Through X-ray diffraction (XRD), the crystallization plane and the ordered structure of the mesoporous silica were observed. The difference between the two types of materials containing MCM-41 and MCM-48 was observed through the different morphologies of the silica particles through scanning electron microscopy (SEM) and also through the Brunauer–Emmet–Teller (BET) analysis, that the surface areas and volumes of pores was different between the two types of mesoporous silica, and, after loading with polyphenols, the values were reduced. The characteristic bands of silica and of polyphenols were easily observed by Fourier-transform infrared spectroscopy (FTIR), and, through thermogravimetric analysis (TGA), the residual mass was determined and the estimated amount of polyphenol in the materials and the efficient loading of mesoporous silica with polyphenols could be determined. The in vitro study was performed in two types of simulated biological fluids with different pH—simulated gastric fluid (SGF) and simulated intestinal fluid (SIF). The obtained materials could be used in various biomedical applications as systems with controlled release of natural polyphenols and the most suitable application could be as food supplements especially when a mixture of such materials is used or when the polyphenols are co-loaded within the mesoporous silica.

## 1. Introduction

Polyphenols are a group of secondary metabolites of plants and are known for their many pharmacological activities due to their ability to interact with molecules in the human body [1,2,3,4,5]. Knowing the clear potential of polyphenols, they represent a considerable interest in the development of dietary supplements [6]. Dietary supplements can contain one or more components in a concentrated state or in combination with other extracts to increase the antioxidant effect [7]. There are limitations in determining the daily intake of polyphenols because this corresponds to the differences between eating behavior and the selection of different food sources [8,9]. In addition, it is well-known that the composition may vary in a large range, depending on many factors. This is why many supplements are developed in trying to get a suitable formula, with proper amount of active components. The low bioavailability of polyphenolic compounds represents one of the major challenge for the administration of supplements, which directs us to the development of new administration systems [8,10]. The bioavailability of polyphenols can be influenced by many factors such as food processing, their interaction with other components, including oxygen, or other factors related to their rapid metabolism in the body [11]. Because of these limitations, an increasing number of formulations based on phenolic acids loaded in different supports are going to be developed in the next few years, these supports acting as protective structures, similar to the specific membranes in the plant structures which keep these components active for a long period of time [12,13].

Caffeic acid, trans-ferulic acid and p-coumaric acid belong to the class of phenolic acids, an important class in the category of polyphenols [14,15,16,17]. These polyphenols are found in food in different concentrations, with caffeic acid generally being the most abundant phenolic acid in the total hydrocinnamic acid content of most fruits [18]. Among the many properties of these phenolic acids, the most important and most studied are antioxidant, anti-inflammatory and anti-coagulatory activities; prevention of oxidative damage/stress; and regulation of gut-microbiota activity [13,19,20,21,22,23]. The contribution of each polyphenol in each of the above-mentioned properties is different. This is why, for a proper diet, diversity is recommended, and why, for a specific deficiency, specific foods are recommended. There are many papers and reports related to the correlation of diet and chronic diseases but these only marginally make reference to polyphenols [24].

The development of nanotechnology has led to the formation of carriers from different materials used as vehicles for the transport of various active substances [25,26,27]. Many studies focus on obtaining controlled release systems (DDSs) so that the transport of nanoparticles is efficient and the active substances are released in the body [28,29]. Nanocarriers based on mesoporous silica nanoparticles (MSNs) have been intensively developed due to their characteristics such as pore size and porosity, biocompatibility, stability and surface functionalization. Mesoporous silica nanoparticles are synthesized in a variety of forms. These allow for the loading of large amounts of biologically active substances (drugs, polyphenols, natural extracts, etc.) and can be directed into the body depending on the target tissues [30,31,32,33,34,35]. Support can be active of a passive, depending on the administration mode. Usually, in contact with the bone, silica-based materials turn into wollastonite and then apatite, which is easily integrated and acts as a support in new bone formation [36]. Mesoporous silica has been reported to be safe for oral administration without significant adverse events or safety concerns being mainly eliminated from the body without transformation [37,38]. Silica-based mesoporous materials are exploited for many applications and this is why, in the last 40 years (since the first research was published in 1992) many improvements have been reported and new classes of mesoporous materials based on silica, including SBA, FSM, TUD, HMM and FDU obtained [32,39,40].

The structure of porous silica nanoparticles allows for their loading with polyphenols and facilitates the controlled release to the target tissues [41,42,43]. In this way, due to the prolonged release of the polyphenols, their biological activity is maintained longer. 

In this study we prepared two types of mesoporous silica with different structures and different pore sizes. We loaded the mesoporous silica nanoparticles by an assisted pore-vacuum methodology with caffeic acid, trans-ferulic acid and p-coumaric acid and characterized the materials obtained by numerous techniques. The obtained systems were characterized from a morphological and structural point of view and the release of polyphenols from mesoporous silica in two biological fluids was studied. Compared with the mesoporous silica loaded with gallic acid described in our previous papers, these polyphenols had higher diversity, thus assuring a larger flexibility. In fact, because of the known the specificity of each polyphenol, by proper combination of the polyphenols, a proper recovery and dose, as well as more equilibrated biological activity, can be assured. 

## 2. Materials and Methods

### 2.1. Materials

**Figure 1:**
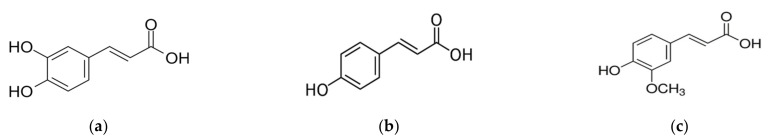
Structure of caffeic acid (**a**), p-coumaric acid (**b**) and trans-ferulic acid (**c**).

Caffeic acid (CA, ≥98%), trans-Ferulic acid (FA, ≥98%), p-Coumaric acid (p-CA, ≥98%), ammonia (NH_3_), tetraethyl orthosilicate (TEOS), ethyl alcohol (absolute), sodium chloride (NaCl) and acetonitrile (HPLC grade) were purchased from Sigma Aldrich and cetyltrimethylammonium bromide (CTAB) from Merck. Sodium hydroxide (NaOH, 1 N) and hydrochloric acid (HCl, 2 N) were purchased from S.C. Silal Trading SRL and kalium dihydrogen phosphate (KH_2_PO_4_, ≥98%) from Roth. Distilled water was used in all experiments in this study. All the substances were used without further purification (Figure 1).

### 2.2. Equipment

The materials-based mesoporous silica and caffeic acid, p-coumaric acid and trans-ferulic acid were characterized using specific physico-chemical methods. 

XRD spectra were recorded on Panalytical X’Pert Pro MPD equipment, with Cu-Kα radiation. 

FTIR analysis was performed using Thermo IN50 MX equipment and an FTIR microscope, operated in reflection mode, was used to study structural features. 

BET analysis was performed on a Micrometrics Gemini V surface area and pore size analyzer. The surface morphology of the samples was examined via a QUANTA INSPECT F electron microscope equipped with a field-emission gun and an energy-dispersive (EDS) detector, on samples covered with silver. 

Thermogravimetric analyses were recorded using a Netzsch 449C STA Jupiter instrument (Mt. Juliet, TN, USA) at room temperature at 900 °C, in an alumina crucible at a heating rate of 10 °C/min in dry air (20 mL/min). 

To study the release of the polyphenols over time, a high performance liquid-chromatography-type Agilent 1260 Infinity with Array Diode Detector (HPLC-DAD) was used. The mobile phase consisted of ultrapure water and acetonitrile, 20/80 (*v*/*v*) for the method in SGF and 30/70 (*v*/*v*) for the method in SIF. Separation was achieved on an Aqua C18 column (250 × 4.6 mm, 5 μm), the flow rate of mobile phase was 0.750 mL/min, and the sample injection volume was 2 μL.

### 2.3. Preparation of the MCM-41 and MCM-48 Mesoporous Materials and Adsorption of Polyphenols

Two mesoporous silica materials types MCM-41 and MCM-48 were obtained using the soft-templating method described in a previous article [44]. The materials were loaded with caffeic acid, ferulic acid and p-coumaric acid by adsorbing the polyphenol solution (saturated solution consisting of polyphenol and acetone) in the pores of the mesoporous material under vacuum at room temperature (Table 1).

### 2.4. In Vitro Release Study

The in vitro release study was performed in two types of simulated biological fluids [45]—simulated gastric fluid (SGF) at pH = 1.2 and simulated intestinal fluid (SIF) at pH = 6.8. The experiments were conducted at a temperature value of 37 ± 1 °C and a stirring speed of 270 rpm. The SGF solution was prepared by dissolving 2 g NaCl in about 1 L deionized water and pH was adjusted to 1.2 with 2 N HCl. The SIF solution was prepared by dissolving 6.8 g KH_2_PO_4_ in about 1 L deionized water and pH was adjusted to 6.8 with 1 N NaOH. For the release study, 50 mg of polyphenol-loaded material was placed in a bag and suspended in a 140 mL bottle of SGF or SIF. Samples were taken at different intervals and absorbance was measured through high-performance liquid chromatography (HPLC).

## 3. Results and Discussions

All the obtained materials were characterized from a structural and morphological point of view so that the difference between the obtained materials and the efficiency of polyphenol loading in the mesoporous silica-based materials could be observed.

**Figure 2:**
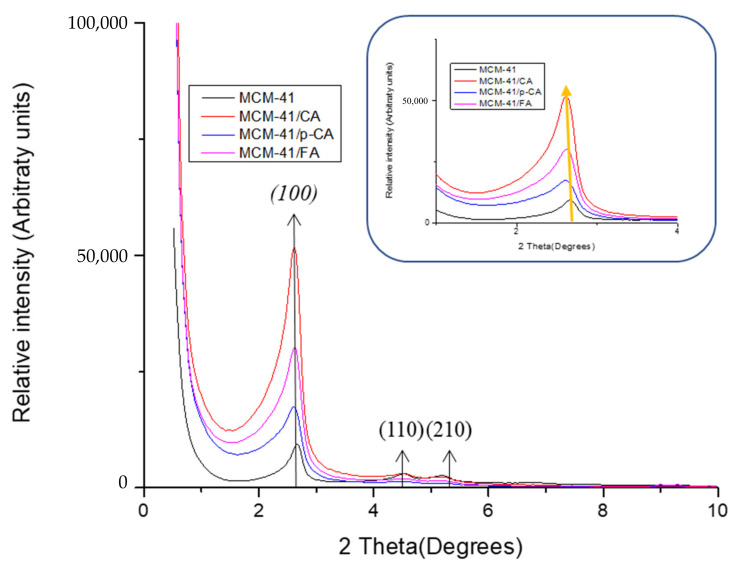
XRD diffractograms of samples MCM-41, MCM-41/CA, MCM-41/p-CA and MCM-41/FA.

All diffractograms recorded on MCM-41 mesoporous materials (Figure 2) show a strong diffraction peak corresponding to the (100) crystallization plane and three other peaks of low intensity attributed to the (110), (200) and (210) crystallization planes feature the ordered hexagonal structure [44]. The ordered mesoporous structure was present in both MCM-41 and polyphenol-loaded mesoporous materials. These peaks are consistent with literature data [46]. In the case of MCM-41 materials loaded with the three polyphenols, a slight shift of the peaks can be observed in the diffractograms of the mesoporous MCM-41/polyphenol materials compared to the characteristic peaks of the MCM-41 material (Figure 2) [47,48]. This behavior can be correlated with the decrease in the pore volume (correlation with BET analysis) as a result of loading the mesoporous material with polyphenols. In conclusion, we can support the fact that a large part of the quantity of polyphenols was loaded into the particles and only a small part remained on the surface of the material. 

**Figure 3:**
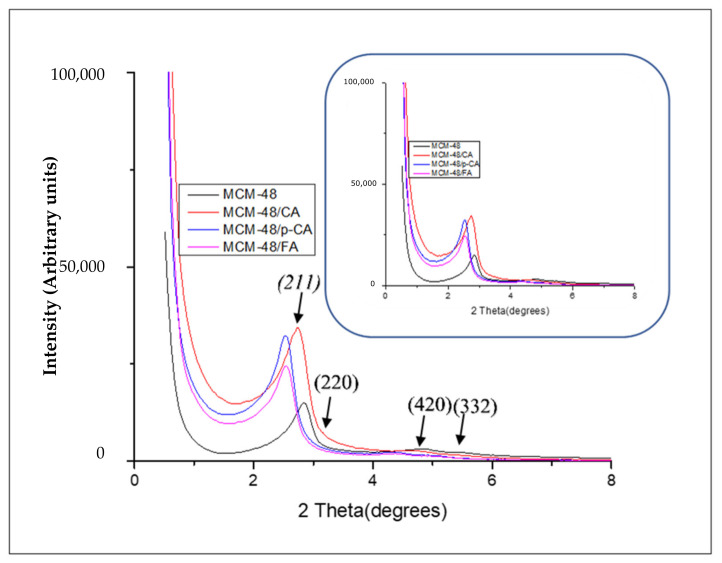
XRD diffractograms of samples MCM-48, MCM-48/CA, MCM-48/p-CA and MCM-48/FA.

The diffractograms recorded on MCM-48 mesoporous materials (Figure 3) show two major peaks corresponding to the crystallization planes (211), (220), (420) and (332). These peaks are consistent with literature data [44]. From the diffractograms recorded on the MCM-48 materials loaded with the three polyphenols, it can be observed that there was a slight shift of the (211) peaks [47,48] due to the loading process. In addition, the characteristic peaks of the (420) and (332) crystallization planes decreased in intensity as a result of the polyphenol adsorption. The decrease in intensity and the displacement of the peaks characteristic of the (211) and (220) crystallization planes, from the diffractograms of the mesoporous MCM-48-type materials loaded with polyphenols, may indicate the loading of the polyphenols into the MCM-48 pores and thus a strong interaction between the two components (Figure 3).

**Figure 4:**
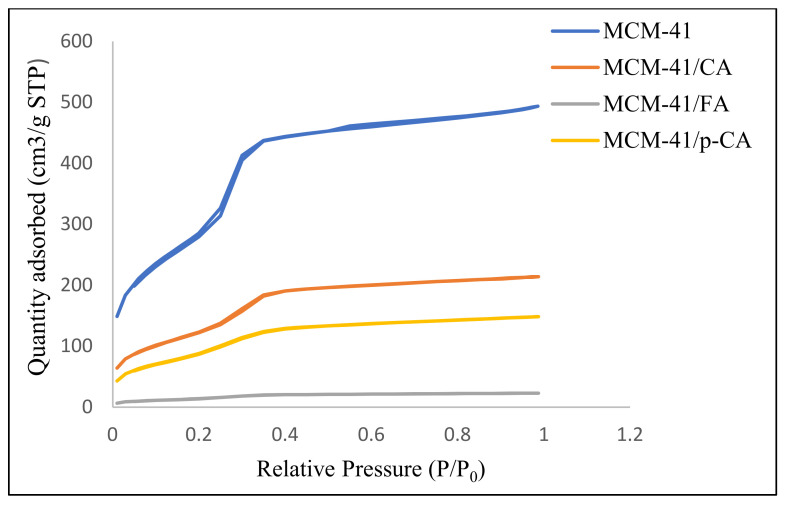
Adsorption isotherms recorded on materials MCM41, MCM-41/CA, MCM-41/p-CA and MCM-41/FA.

All MCM-41 samples loaded with polyphenols showed type IV isotherms showing a hysteresis loop of type H1, characteristic for MCM-41-type mesoporous materials with an ordered hexagonal structure with cylindrical pores of similar sizes [49]. Nitrogen adsorption/desorption isotherms (Figure 4) for the mesoporous materials MCM-41 and MCM-41/CA, MCM-41/p-CA and MCM-41/FA, in the range of relative pressures (p/p^0^) 0.01–0.99, are completely reversible, indicating the uniformity of unidirectional tubular mesopores, characteristic of ordered mesoporous structures [50]. This leads to the conclusion that the adsorption of polyphenols does not change the ordered structure of the pores.

The high specific surface area of MCM-41 of 1179 m^2^/g decreased with the loading of polyphenols, which means that polyphenols were largely absorbed in the pores and, perhaps, only a small amount was deposited on the surface. Similar behavior was obtained for the MCM-48 mesoporous support, as presented in Table 2. As a consequence of the loading, the BET surface area decreased by 2.45 times for the MCM-41/CA, 3.42 times for the MCM-41/p-CA and 21.52 times for the MCM-41/FA. In case of MCM-48, as a consequence of the loading, the BET surface area decreased 1.85 times for the MCM-48/CA, 2.56 times for the MCM-48/p-CA and 3.06 times for the MCM-48/FA.

**Figure 5:**
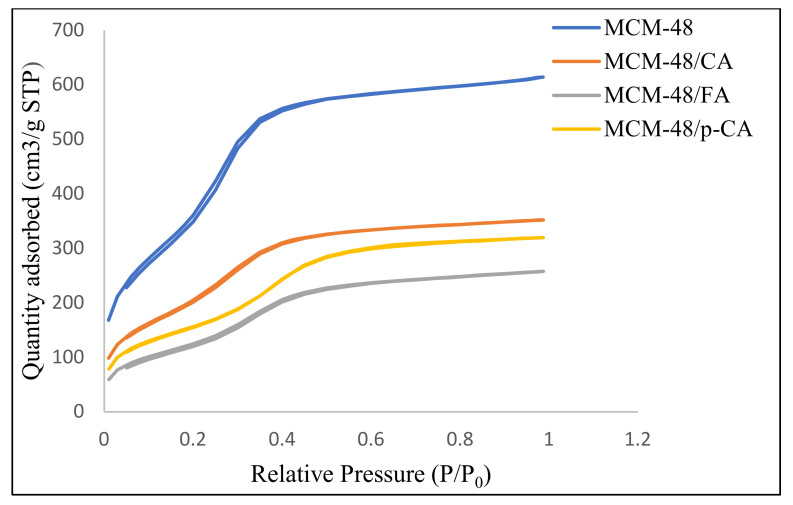
Adsorption isotherms recorded on materials MCM418, MCM-48/CA, MCM-48/p-CA and MCM-48/FA.

The nitrogen adsorption/desorption isotherms (Figure 5) of the mesoporous materials MCM-48, MCM-48/CA, MCM-48/p-CA and MCM-48/FA, in the range of relative pressures (p/p^0^) 0.01–0.99, show type IV isotherms and show a stage of condensation in the relative pressure range of 0.2–0.3 which is correlated with capillary condensation in the pores of mesoporous materials.

**Figure 6:**
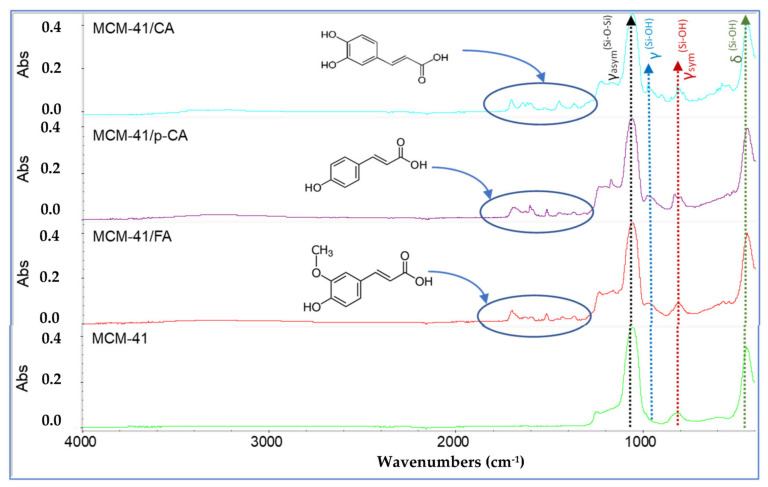
FTIR spectra of samples MCM-41, MCM-41/CA, MCM-41/p-CA and MCM-41/FA.

In conclusion, corroborating the two analyzes, XRD and BET, we can assume that polyphenols are, most likely and to a large extent, adsorbed inside the pores. In the FTIR spectra of the mesoporous materials MCM-41, MCM-41/CA, MCM-41/p-CA and MCM-41/FA (Figure 6), the main characteristic bands of the silica network can be easily observed. The bands at 1243.181 cm^−1^ and 1049.86 cm^−1^ can be assigned to the stretching vibrations of the asymmetric Si-O-Si units, the band at 439.58 cm^−1^ can be assigned to the deformation vibrations of the Si-O-Si units, the short band at 811.48 cm^−1^ can be assigned to the stretching vibrations of the symmetric Si-O-Si units, and the band at ~960–980 cm^−1^ is associated with silanol groups of MCM-41 [44]. 

In the case of the FTIR spectra recorded on the MCM-41/CA-, MCM-41/p-CA- and MCM-41/FA-type materials (Figure 6), a shift of the main characteristic bands of the silica network can be observed. In addition, the bands characteristic of organic functional groups in the structure of absorbed polyphenols can also be observed. From the FTIR spectrum, it can be seen that upon loading the mesoporous material MCM-41 with polyphenols, a series of new bands appear that demonstrate the efficiency of loading with caffeic acid, p-coumaric acid and trans-ferulic acid. Thus, bands in the area of ~3280 cm^−1^ (MCM-41) characteristic of the stretching vibrations of the O–H bonds corresponding to the phenolic groups in the polyphenol structure can be visualized in the IR spectra. In addition, the bands between 1730 cm^−1^ and 1618 cm^−1^ correspond to the stretching vibrations of the OH and C=O groups. The bands at ~1600 cm^−1^ and ~1370 cm^−1^ correspond to the stretching vibrations of the bond between the benzene nucleus and the carboxyl group in the molecule of polyphenol. In addition, the C-H vibrations of the methylene groups in the benzene rings of caffeic, coumaric and ferulic acid can be found in the range of 3000 and 2800 cm^−1^. By comparing the FTIR spectra of the mesoporous material synthesized and modified with polyphenols, it can be observed that the broad bands between ~3200–3400 cm^−1^ (MCM-41) corresponding to the associated OH groups in the polyphenols (O-H stretching vibration) become increasingly pronounced due to the increase in the organic-compound concentration (Figure 6). 

**Figure 7:**
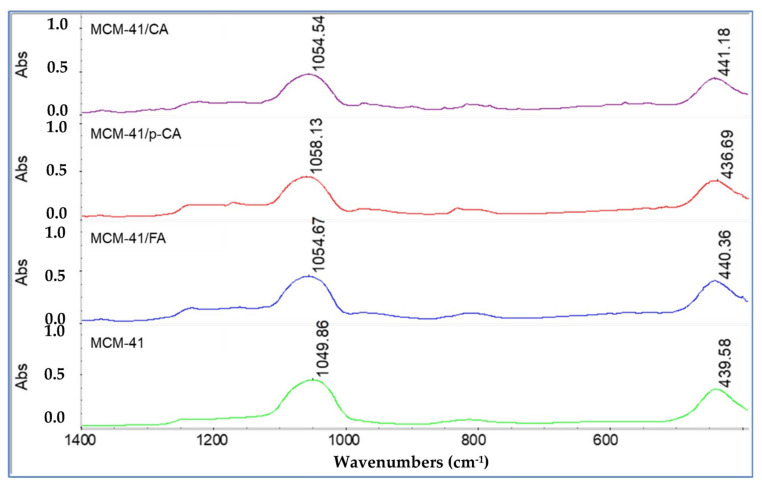
FTIR spectra of samples MCM-41, MCM-41/CA, MCM-41/p-CA and MCM-41/FA (visualization on the 400–1400 cm^−1^ range).

The loading of the mesoporous material MCM-41 with p-coumaric acid induces significant shifts of the MCM-41 characteristic band from 1049.86 and 439.58 cm^−1^ to 1058.13 and 436.69 cm^−1^ (i.e., 8.27 and 2.47 cm^−1^, respectively) (Figure 7).

In the case of MCM-41/CA and MCM-41/FA, these shifts are less important, i.e., the shift occurs from 1049.86 and 439.58 cm^−1^ to 1054.54 and 441.18 cm^−1^ (i.e., 4.68 and 1.6 cm^−1^) and, respectively, from 1049.86 and 439.58 cm^−1^ to 1054.67 and 440.36 cm^−1^ (i.e., 4.81 and 0.78 cm^−1^). These less-important displacements can be explained by a lower interaction of caffeic and trans-ferulic acids with the mesoporous matrix.

**Figure 8:**
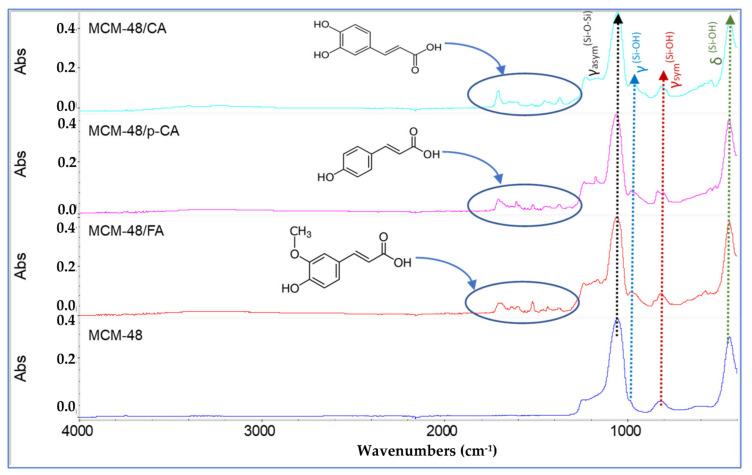
FTIR spectra of samples MCM-48, MCM-48/CA, MCM-48/p-CA and MCM-48/FA.

In the FTIR spectra obtained for the mesoporous material of MCM-48 type (Figure 8), the main characteristic bands of the silica network can be observed. The bands from 1050.35 cm^−1^ and 1241 cm^−1^ are characteristic of the tensile vibrations of the asymmetric O-Si-O units, the band from 436.91 cm^−1^ is characteristic of the deformation vibrations of the O-Si-O units, the band from 811.37 cm^−1^ is characteristic of the tensile vibrations of the symmetrical Si-O-Si units, and the band from ~510 cm^−1^ is characteristic of the deformation vibrations of the symmetrical O-Si-O units. The band from 982.50 cm^−1^ is associated with the silanol groups of MCM-48. In the case of the IR spectra recorded on the MCM-48/CA-, MCM-48/p-CA- and MCM-48/FA-type materials (Figure 8), we can observe, from the structure of absorbed polyphenols, that the main characteristic bands of the silica network as well as the bands characteristic of organic functional groups, shifted to longer wavelengths. From the FTIR spectra recorded on the three materials, it can be seen that after loading the MCM-48 material with polyphenols, a series of new bands appeared that demonstrate the efficiency of loading with caffeic acid, p-coumaric acid and ferulic acid.

**Figure 9:**
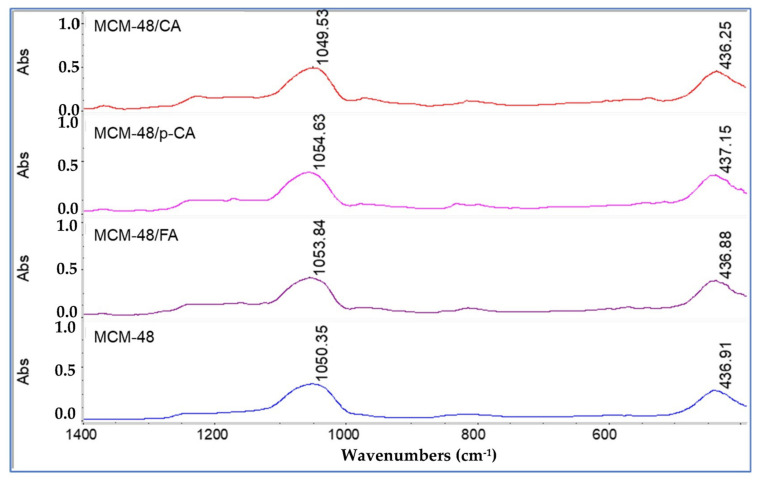
FTIR spectra of samples MCM-48, MCM-48/CA, MCM-48/p-CA and MCM-48/FA (visualization on the 400–1400 cm^−1^ range).

As in the case of the MCM-48 material, the loading of the MCM-48 mesoporous material with the three polyphenols induces changes in the MCM-48 characteristic band from 1050.35 cm^−1^ and 436.91 cm^−1^ (Figure 9) at 1049.53 cm^−1^ and 436.25 cm^−1^ (i.e., 0.82 cm^−1^ and 0.68 cm^−1^) in the case of MCM-48 loading with caffeic acid; at 1054.63 cm^−1^ and 437.15 cm^−1^ (i.e., 4.28 cm^−1^ and 0.24 cm^−1^) in the case of MCM-48 loading with p-coumaric acid; and from 1050.35 cm^−1^ and 436.91 cm^−1^ to 1053.84 cm^−1^ and 436.88 cm^−1^ (i.e., 3.49 cm^−1^ and 0.03 cm^−1^) in the case of MCM-48 loading with ferulic acid, respectively. These smaller shifts can be explained by a lower interaction of polyphenols with the mesoporous matrix.

**Figure 10:**
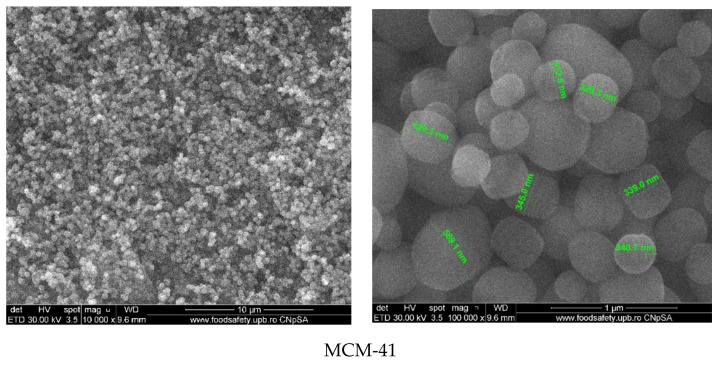

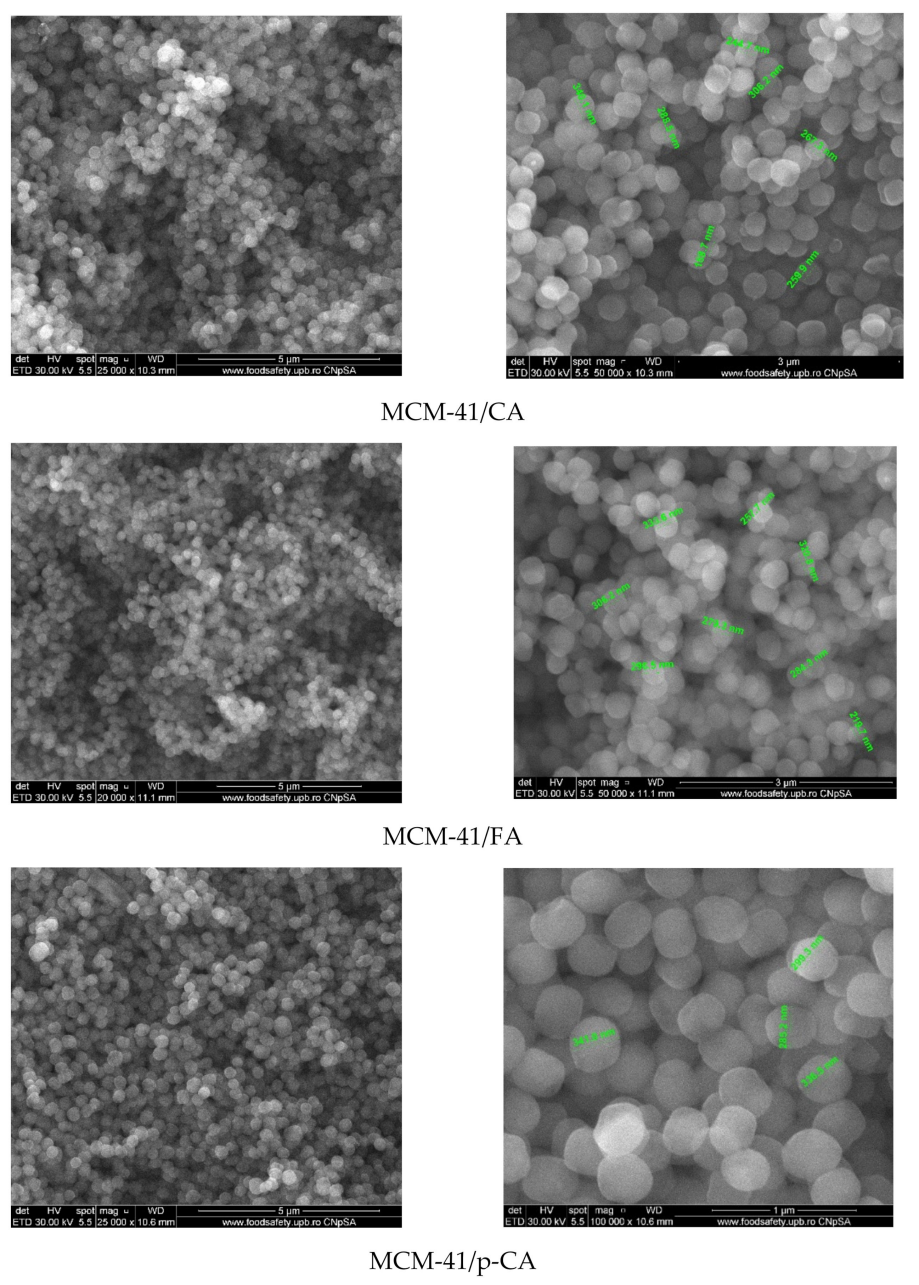
SEM images of MCM-41 mesoporous materials loaded with polyphenols.

Scanning-electron-microscopy images (Figure 10) of MCM-41 mesoporous material show spherical morphology of silica particles with variable sizes in the range of 150–400 nm. Compared to MCM-41, SEM images of MCM-41/polyphenols show some heterogeneous areas in the form of agglomerates, which can be attributed to the presence of polyphenols. These agglomerates are much smaller compared to the spherical particles of MCM-41, and their content is low, which means that only a small part of the polyphenol was deposited on the surface of MCM-41. This was mostly arranged inside the pores. At high magnification (100,000×), it could be clearly observed that the SEM images of the MCM-41 sample are more translucent than those of the samples loaded with polyphenols. These observations are in good agreement with the BET analyses.

**Figure 11:**
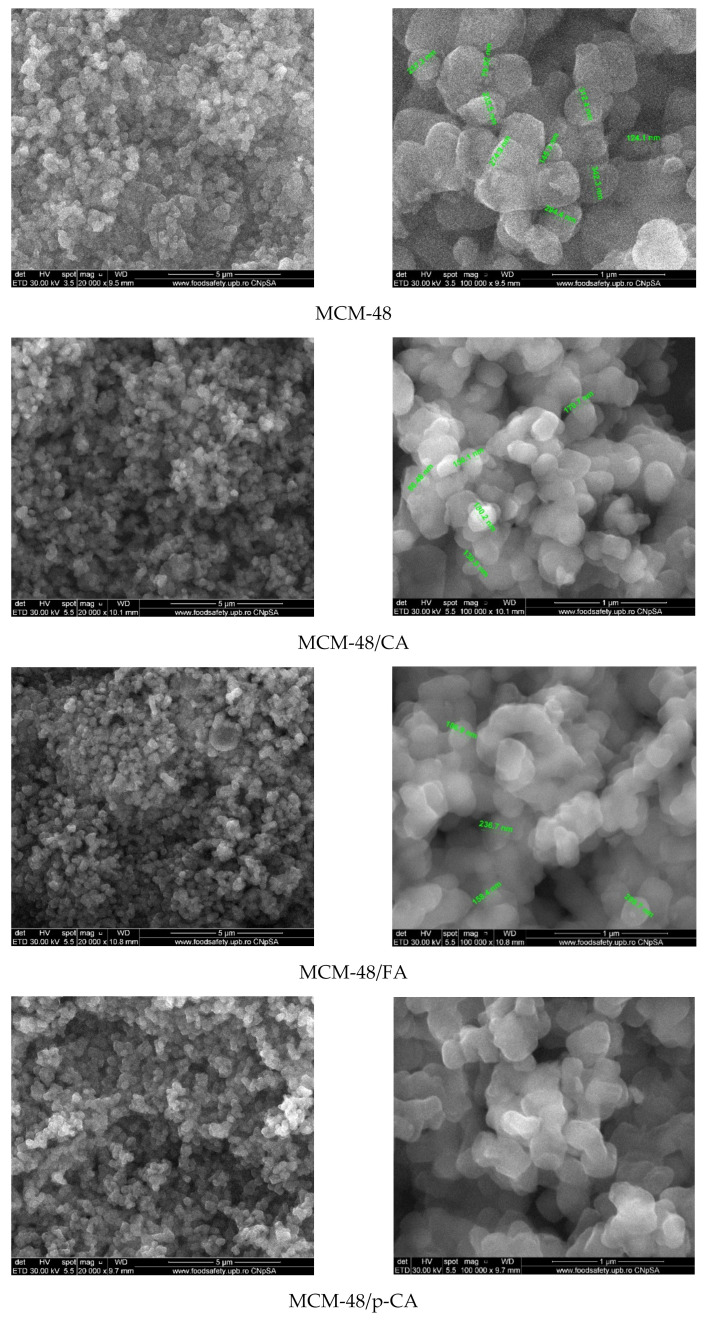
SEM images of MCM-48 mesoporous materials loaded with polyphenols.

Scanning electron microscopy images recorded on MCM-48 mesoporous material (Figure 11) show the quasi-spherical morphology of silica particles with varying sizes in the range of 150–400 nm. Compared to MCM-48, the SEM images of MCM-48/polyphenols) show some heterogeneous areas in the form of agglomerates, which can be attributed to the presence of polyphenols but there were no significant changes such as strong agglomerations or entrapment of the mesoporous materials within the polyphenolic compounds. 

**Figure 12:**
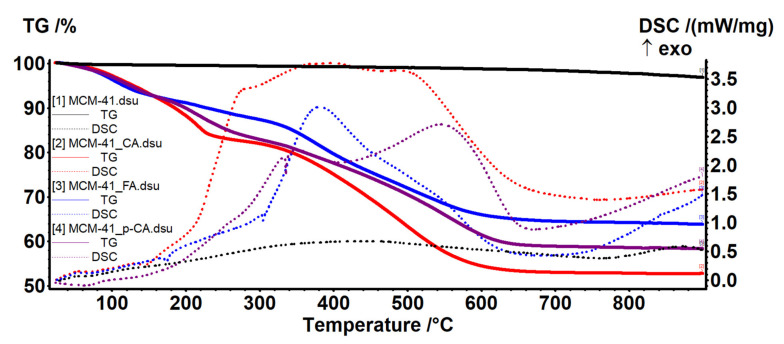
TG–DSC curves for MCM-41, pristine and loaded with polyphenols.

The thermal stability of the samples, both those consisting of mesoporous materials and those materials loaded with polyphenols, was studied by thermogravimetric analysis (Figure 12).

The amount of adsorbed water and the quantity of silanol groups on the surface of MCM-41 sample was evaluated as indicated in [44]. The literature indicates two temperature intervals for elimination of adsorbed water molecules (RT–200 °C) and for condensation of silanol groups (200–900 °C). Thus, between RT and 200 °C the sample is losing 1.86% of its initial mass, represented by the physically adsorbed water molecules, from both surface and inside the pores. As such, up to ~100 °C the sample is losing the unbound water, which filled the pores with little or no surface interactions (0.50%). In the temperature interval 100–200 °C water molecules that interact with the surface of MCM-41, and therefore require higher energies to break free (1.36%) were eliminated. The release of these two types of water molecules is indicated by the endothermic effects on DSC curve, with minimums at 64.1 and 188.5 °C [51].

In the temperature interval 200–900 °C the water is eliminated by condensation of silanol moieties, the process being responsible for silica formation and densification. The process was slow, with low intensity, giving the continuous-mass-loss aspect of the TG curve in this interval (2.60%). The recorded residual mass at 900 °C was 95.53%. The most important data are summarized in the Table 3.

The densities of H_2_O molecules and –OH moieties were calculated as indicated in [44]. The amount of H_2_O molecules and –OH moieties per 1 g MCM-41 is expressed as n_H2O_ and n_OH_ and calculated with formula from Equation (1):

n_OH_ = 2n_H2O_ = 2 (W_T0_ − W_Tfin_)/(100 ∗ M_H2O_) (1)

where W_T0_ − W_Tfin_ is the weight loss (wt.%) in temperature range T_0_ − T_fin_ and M_H2O_ is the water molecular mass.

The number of water molecules/-OH groups (N_H2O_; N_OH_) per 1 nm^2^ was calculated from Equation (2):

N = n ∗ N_A_ ∗ 10^−18^/S, (2)

where n is the amount of water/-OH groups (mmol/g), N_A_ is Avogadro number and S is the specific surface area of sample from BET.

**Figure 13:**
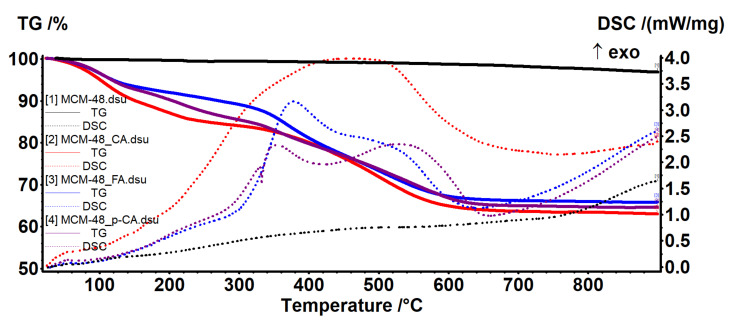
TG–DSC curves for MCM-48, pristine and loaded with polyphenols.

The amount of adsorbed water and the quantity of silanol groups on the surface of MCM-48 sample was evaluated in a similar way (Figure 13). Between RT and 200 °C the sample lost 0.97% of its initial mass, represented by the physically adsorbed water molecules, from both surface and inside the pores. This process was accompanied by a weak endothermal effect on the DSC curve, with minimum at 68.7 °C. In the temperature interval 100–200 °C the water molecules that interact with the surface of MCM-48, and therefore require higher energies to break free (0.54%), were eliminated.

We recorded a mass loss of 1.97% in the temperature interval 200–900 °C, represented by condensation of silanol moieties. The process was slow, with low intensity, giving the continuous mass loss aspect of the TG curve in this interval (1.97%). The residual mass at 900 °C was 97.15%.

The polyphenol-loaded samples exhibited larger mass losses, which can be grouped in three intervals. Between RT and 150 °C the elimination of free water molecules and the start of degradation of the adsorbed organic molecules took place. The adsorbed organic molecules interacted with silica support, causing the second endothermic effect visible on the DSC curve. The degradation of the polyphenols continued slowly between 150 and 340 °C. After 340 °C a strong oxidative processes took place leading to the burning of the organic residue; this process was accompanied by large exothermic effects on the DSC curves. By comparing the residual mass at 900 °C for the pristine silica support and the polyphenol-loaded samples, we can estimate the amount of organic substances loaded (Table 4).

**Figure 14:**
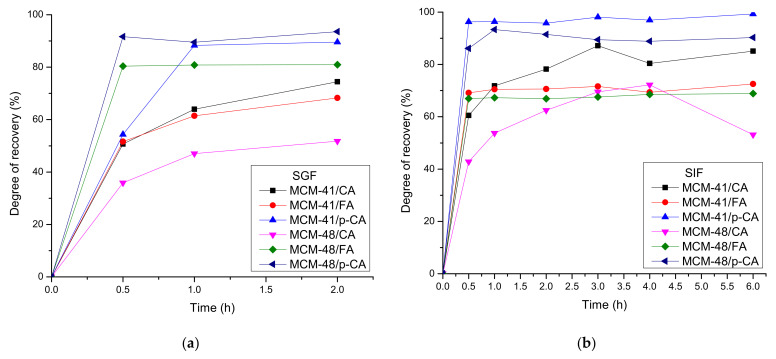
Degree of recovery from mesoporous silica support types MCM-41 (**a**) and MCM-48 (**b**).

In Figure 14 represents the release profiles of polyphenols from MCM-41 and MCM-48 mesoporous silica supports in two simulated biological fluids, SGF and SIF. The releases were monitored for 2 h in SGF and 6 h in SIF to observe the different release profiles of the polyphenols.

Comparing the release profiles of caffeic acid from the two mesoporous silica supports, a major difference can be observed between the release curves of MCM-41/CA and MCM-48/CA. The behavior of both types of materials in the two biological fluids was similar, observing in SGF a degree of recovery for MCM-41/CA of ~70%, unlike MCM-48/CA in which a degree of recovery was of ~50%.

The degree of recovery of ferulic acid from the mesoporous silica supports was over 50% in the first 30 min, in both biological fluids. The values of the degrees of recovery were maintained throughout the release study with the exception of the degree of recovery of ferulic acid from MCM-41 in SGF in which an increase of up to ~68% in 2 h was observed. Comparing the release profiles in the two biological fluids, it was observed that the degree of recovery of ferulic acid from MCM-48/FA in SGF was higher than in SIF.

Unlike ferulic acid and caffeic acid, the release profile of p-coumaric acid was fast— the degree of recovery of p-coumaric acid from MCM-41 and MCM-48 was over 90% after the first hour, and this was maintained throughout the release study. Thus, in Figure 14b, the release profile of the MCM-41/p-CA sample in SIF was the fastest and highest, unlike the other samples, the lowest being that of the MCM-41/FA sample in SIF. In Figure 14a, the MCM-48/p-CA sample had the fastest and highest release profile in SGF.

Observing all the release profiles of the polyphenols from the mesoporous supports we can see that there were differences in relation to three important factors: the polyphenols due to their different structures and thus interactions with the support, the two biological fluids due to the different pH, and the two types of mesoporous silica supports having a different pore structure [52,53,54]. These differences can be mainly associated with the different solubility of the polyphenols and their different interactions with the pores of the supports (with the surface). It is important to mention that depending on the pH, the phenolic and the carboxylic acid groups can be ionized differently and also the SiOH:SiO^−^Na^+^ groups on the surface of the silica supports will be changed. Moreover, the interaction between the polyphenols and the silica surface will be different. Depending on the pore size, the solvent diffusion and the polyphenol release will be different too. A well-known problem with the organic acids is related to their solubility, which is strongly influenced by the pH. Considering the existing concerns related to the use/consumption of the polyphenols, especially as pure compounds (not as foods), there are some concerns related to their overconsumption [28,29,55]. Such porous materials loaded with polyphenols can assure a day-long release of these agents and in this way mimic the release (availability) from natural foods. Considering the antioxidant, anti-inflammatory, antithrombotic, antimicrobial and anti-carcinogenic activity of these polyphenols [56,57,58] as well as the release data of the three polyphenols, further studies will be designed by mixing these polyphenols in specific ratios in order to tune these properties, for in vitro and in vivo assays.

## 4. Conclusions

In this study, two types of mesoporous silica, MCM-41 and MCM-48, were synthesized. The results of the XRD and BET analyzes showed that two types of mesoporous materials were obtained with different silica structure and pore size. To observe the loading method of mesoporous silica, we loaded both types of mesoporous silica with three polyphenols: caffeic acid, p-coumaric acid and ferulic acid. Combining the results of BET, FTIR and thermogravimetric analyses, we can conclude that polyphenols were loaded inside the pores of mesoporous silica, and this is why the BET surface area decreased from 1179 down to 54 m^2^/g (for the MCM-41 sample loaded with ferulic acid) and from 1482 down to 482 m^2^/g (for the MCM-48 sample loaded with the same ferulic acid). Depending on the nature of the polyphenol, but also on the type of mesoporous silica, important differences, such as pore structure, pore loading capacity and surface morphology were obtained. In order to study and observe the applications of the obtained systems, we performed release studies in two types of biological fluids, SGF and SIF. In these release studies, different release profiles were obtained, which proves that these systems can be used in different applications. The degree of recovery of polyphenols varied between 70 and ~100%, the steady state being obtained within 6 h in SIF. Even if the release of the same polyphenol from the same support was quite independent of the simulated fluid used, the three polyphenols exhibited important differences from the point of view of cumulative release. It is expected that a proper combination of the polyphenols could assure the proper level of polyphenols and that this could be tuned to the desired activity. In conclusion, the systems obtained from mesoporous silica and polyphenols (caffeic acid, p-coumaric acid and trans-ferulic acid) could be used in various biomedical applications, especially as food supplements, but additional studies are needed to prove their applicability.

## Figures and Tables

**Table 1:** Type of polyphenol-loaded mesoporous materials.

No.	Material Type	Mass Ratio Mesoporous Material:Active Substance (*w*:*w*)
MCM-41	MCM-41	
MCM-41/CA	MCM-41: Caffeic acid	1:0.4
MCM-41/FA	MCM-41: Ferulic acid	1:0.4
MCM-41/p-CA	MCM-41: p-Coumaric acid	1:0.4
MCM-48	MCM-48	
MCM-48/CA	MCM-48: Caffeic acid	1:0.4
MCM-48/FA	MCM-48: Ferulic acid	1:0.4
MCM-48/p-CA	MCM-48: p-Coumaric acid	1:0.4

**Table 2:** BET characteristics of mesoporous materials.

Type of Material	BET Surface Area m^2^/g	Langmuir Surface Area m^2^/g	Volume of Pores cm^3^/g
MCM-41	1179.6395	1765.7942	0.638326
MCM-41/CA	480.6665	713.4224	0.271356
MCM-41/FA	54.8102	82.0561	0.026724
MCM-41/p-CA	344.9734	515.5435	0.172790
MCM-48	1482.9160	2250.0934	0.748646
MCM-48/CA	803.3241	1203.8926	0.421469
MCM-48/FA	482.5568	719.3688	0.340084
MCM-48/p-CA	578.6620	852.0359	0.414938

**Table 3:** MCM-41 and MCM-48 characteristics from the TGA and BET values.

Sample	Mass Loss % RT–200 °C	Mass Loss % 200–900 °C	Endo Effect (°C)	Residual Mass % (900 °C)	n_H2O_ (mmol/g)	n_OH_ (mmol/g)	N_H2O_ (Groups/nm^2^)	N_OH_ (Groups/nm^2^)
MCM-41	1.86	2.60	64.1	95.53	1.03	2.89	0.53	1.48
MCM-48	0.97	1.97	68.7	97.15	0.54	2.19	0.22	0.89

**Table 4:** Estimated polyphenol quantities loaded on MCM-41 and MCM-48.

Sample	Residual Mass, %	Estimated Polyphenol Load, %
MCM-41/CA	52.76	44.77
MCM-41/FA	63.86	33.15
MCM-41/p-CA	58.35	38.92
MCM-48/CA	63.04	35.11
MCM-48/FA	65.81	32.26
MCM-48/p-CA	64.53	33.58

## Data Availability

These data can be available upon request!
